# Infrared stimulation of mitochondrial metabolism in deep-sea yeast expands the energetic framework of hydrothermal ecosystems

**DOI:** 10.1093/ismeco/ycag129

**Published:** 2026-05-19

**Authors:** Jie Dai, Luyao Zhang, Yingshan Li, Rui Zhao, Xue-Gong Li, Jihong Liu, Artemis Kosta, Hugo Le Guenno, Salomé Sauvage, Wei-Jia Zhang, Régine Lebrun, Yue Shen, Huanming Yang, Changbin Chen, Long-Fei Wu

**Affiliations:** Aix Marseille University, CNRS, LCB, IMM, IM2B, CENTURI, Marseille 13402, France; Research Centre of Ecology & Environment for Coastal Area and Deep Sea, Southern Marine Science and Engineering Guangdong Laboratory (Guangzhou), Guangzhou 511458, China; The Joint Laboratory for Biomedical Research and Pharmaceutical Innovation, Unit of Pathogenic Fungal Infection & Host Immunity, Shanghai Institute of Materia Medica, Chinese Academy of Sciences, Shanghai, China; BGI Research, Changzhou 213100, China; Laboratory of Deep-Sea Microbial Cell Biology, Institute of Deep-sea Science and Engineering, Chinese Academy of Sciences, Sanya 572000, China; Laboratory of Deep-Sea Microbial Cell Biology, Institute of Deep-sea Science and Engineering, Chinese Academy of Sciences, Sanya 572000, China; CAS Key Laboratory for Experimental Study under Deep-sea Extreme Conditions, Institute of Deep-sea Science and Engineering, Chinese Academy of Sciences, Sanya 572000, China; Institution of Deep-sea Life Sciences, IDSSE-BGI, Hainan Deep-sea Technology Laboratory, Sanya 572000, China; The Joint Laboratory for Biomedical Research and Pharmaceutical Innovation, Unit of Pathogenic Fungal Infection & Host Immunity, Shanghai Institute of Materia Medica, Chinese Academy of Sciences, Shanghai, China; Microscopy Core Facility, FR3479 IMM, CNRS, Aix Marseille University, Marseille 13000, France; Microscopy Core Facility, FR3479 IMM, CNRS, Aix Marseille University, Marseille 13000, France; Marseille Protéomique, IMM, CNRS, Aix Marseille University, Marseille 13000, France; Laboratory of Deep-Sea Microbial Cell Biology, Institute of Deep-sea Science and Engineering, Chinese Academy of Sciences, Sanya 572000, China; CAS Key Laboratory for Experimental Study under Deep-sea Extreme Conditions, Institute of Deep-sea Science and Engineering, Chinese Academy of Sciences, Sanya 572000, China; Institution of Deep-sea Life Sciences, IDSSE-BGI, Hainan Deep-sea Technology Laboratory, Sanya 572000, China; Marseille Protéomique, IMM, CNRS, Aix Marseille University, Marseille 13000, France; BGI Research, Changzhou 213100, China; Guangdong Provincial Key Laboratory of Genome Read and Write, BGI Research, Shenzhen 518083, China; Guangdong Provincial Key Laboratory of Genome Read and Write, BGI Research, Shenzhen 518083, China; The Joint Laboratory for Biomedical Research and Pharmaceutical Innovation, Unit of Pathogenic Fungal Infection & Host Immunity, Shanghai Institute of Materia Medica, Chinese Academy of Sciences, Shanghai, China; Aix Marseille University, CNRS, LCB, IMM, IM2B, CENTURI, Marseille 13402, France

**Keywords:** deep-sea hydrothermal vents, blackbody emission, geothermal infrared radiation, chemosynthesis, photosynthesis, radiative energy

## Abstract

Deep-sea hydrothermal vents are archetypal chemosynthetic ecosystems, yet the intense geothermal near-infrared (NIR) radiation emitted by black smoker chimneys remains a largely unexplored environmental factor. While infrared (IR)-driven photosynthesis has been described in vent-associated bacteria, whether deep-sea eukaryotes can biologically respond to geothermal NIR radiation has remained unknown. Here, we demonstrate that the vent yeast *Rhodotorula mucilaginosa* F-J1 exhibits wavelength-specific growth stimulation under NIR exposure through a previously unrecognized mitochondria-centered metabolic response. NIR exposure significantly enhanced growth beyond the effects of glucose supplementation and reduced the apparent activation energy for growth, consistent with enhanced cellular metabolism. Mechanistically, targeted inhibition and ultrastructural analyses identified mitochondrial oxidative phosphorylation and mitochondrial ribosomal activity as essential components of this response. Proteomic and heterologous expression analyses further identified the mitochondrial ribosomal protein MRPS18b as a key mediator of NIR-stimulated growth. These findings reveal a previously unrecognized capacity of deep-sea eukaryotic microbes to biologically respond to geothermal IR radiation and expand the known energetic framework of hydrothermal vent ecosystems beyond canonical chemosynthesis and photosynthesis.

## Graphical Abstract

**Graphical Abstract:**
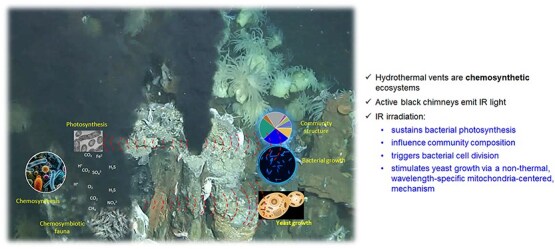

## Introduction

Deep-sea hydrothermal vents rank among the most energy-rich yet light-limited environments on Earth. At these sites, hot, chemically reduced fluids emerge from the seafloor and fuel microbial primary production via chemosynthesis [1]. This paradigm has long characterized vent ecosystems as fully chemosynthetic—supported exclusively by chemical energy. However, black smoker chimneys, which reach temperatures of ~400°C, also emit intense infrared (IR) radiation through blackbody emission [2, 3]. Previous studies have demonstrated that IR-absorbing chlorophyll pigments sustain photosynthesis in deep-sea bacteria [4, 5]. More recently, sulfide mineral–mediated upconversion of IR to visible light has been shown capable of supporting photosynthesis [6]. Collectively, these findings suggest that geothermal infrared radiation represents a previously overlooked energy source in the deep sea—consistent with the hypothesis that geothermal light may have played a role in the early evolution of photosynthesis [7, 8]. Thus, hydrothermal vents may support not only chemosynthetic but also photosynthetic strategies.

Our previous work provided experimental support for the hypothesis that infrared radiation can serve as an environmental cue for prokaryotic life. Metagenomic analyses of a hydrothermal vent on the Southwest Indian Ridge (SWIR) revealed diverse phototrophic bacterial lineages, complete chlorophyll biosynthesis pathways, and both blue- and green-light-absorbing bacteriorhodopsins [9]. Subsequent IR enrichment experiments demonstrated that IR exposure significantly alters microbial community composition and stimulates bacterial growth [10]. In addition, in the vent bacterium *Tepidibacter hydrothermalis* SWIR-1 [11], high hydrostatic pressure inhibited cell division, whereas IR irradiation restored growth by reinitiating peptidoglycan synthesis [10]. However, whether eukaryotic organisms—abundant and ecologically significant members of hydrothermal systems—can similarly exploit geothermal IR radiation remains unknown. Addressing this question is critical for understanding how radiative energy flux contributes to the broader energetic landscape of deep-sea ecosystems.

To investigate this, we isolated a deep-sea yeast, *Rhodotorula mucilaginosa* strain F-J1, from a hydrothermal vent field in the Central Indian Ocean. We characterized its physiological and molecular responses to near-infrared (NIR; 810–1050 nm) radiation. Our results demonstrate that NIR irradiation stimulates yeast growth, accompanied by a significant reduction in activation energy and enhanced mitochondrial energy metabolism. We identify a lineage-specific mitochondrial ribosomal protein, MRPS18b, as a key mediator of this NIR-stimulated growth, providing evidence for a previously unrecognized biological response pathway to geothermal NIR exposure in the deep sea.

## Materials and methods

### Sample collection and isolation of infrared boosted strains

During the TS10-3 expedition in February 2019, the manned submersible *Shen Hai Yong Shi* was used in dive SY134 to survey the Edmond hydrothermal vents in the Central Indian Ocean. Sediment samples were collected from a site covered by a shrimp swarm 0.5 m away from an active black chimney at ~378°C located at 69.60°E, 23.88°S, at a depth of 3310 m. The temperature at the sampling site was ~10°C. Once onboard, the samples were preserved at 4°C as described previously [9, 11, 12].

Upon reaching the laboratory on land, a 2 g sample was resuspended in 10 ml of artificial seawater (For composition, see Supporting information (SI)). Then, 100 μl of the suspension was inoculated into 3 ml of 2216 marine broth (see SI), or H9-0.5 medium (see SI) in 10 ml plastic culture tubes. These were incubated at 26°C in the dark or under 880 nm infrared irradiation at 3–4 mW, measured using a Thorlabs power meter (PM100D) equipped with an S120C sensor, positioned at the level of the surface of the culture tubes. The 880 nm NIR was chosen because irradiation at this wavelength effectively influenced population composition and stimulated bacterial growth without a significant heating effect [10].

The optical density at 600 nm of the cultures was measured using Cary-60 (Agilent, China or France) after varying incubation periods ranging from one to several days. Cultures that appeared to be enriched by NIR irradiation were diluted 1:100 in fresh corresponding media and then re-incubated either in the dark or under 880 nm NIR irradiation. The IR-enriched cultures were spread (100 μl each) onto 2216 marine broth or H9-0.5 media plates, which were subsequently incubated at 26°C. Colonies that formed on these plates were further characterized.

### Physiological characterization of strain F-J1

F-J1 was cultured in YPD (1% yeast extract, 2% peptone, 2% glucose), GYPS (0.1% yeast extract, 0.1% peptone, 0.1% glucose, 0.1% starch, 2% NaCl), GYP (0.1% yeast extract, 0.1% peptone, 0.1% glucose, 0.1% starch), or YPS (0.1% yeast extract, 0.1% peptone, 2% NaCl). For routine growth assays, the OD_600_ of the cultures was measured using Cary-50 (Agilent, France), the cell number was calculated with a hemocytometer under a microscope. To investigate the effects of temperature and NIR irradiation on F-J1 growth, cells from overnight precultures were inoculated at a 1:100 dilution into fresh GYPS media. The inoculum was then distributed into four 3 ml quartz cuvettes, with 1.8 ml per cuvette. Cultivation and absorption spectra analyses were performed using a Turret 4 Temperature-Controlled Cuvette Holder from Quantum Northwest Inc. USA (www.qnw.com), integrated into a Cary 60 spectrophotometer (Agilent, France). The temperature was rigorously maintained across all four cuvettes using a Peltier thermal controller and a water circulation system connected to a thermostat set at the defined temperature. Temperature stability was managed via a TC1 Temperature Controller and monitored in real time using a QNW Probe/10 K micro-thermistor probe (Fig. S1). Gas flushing was employed to remove condensation water around the cuvettes during low-temperature experiments. To closely mimic the conditions of the *in situ* sampling site, the cultures were not stirred. For comparison, two *R. mucilaginosa* strains, DSM 13621 and DSM 18184, were purchased from DSMZ-German Collection of Microorganisms (https://www.dsmz.de/collection/catalogue). *Saccharomyces cerevisiae* W303 was a gift from Y. Corda [13].

Five Thorlabs light-emitting diodes (LEDs) (Thorlabs, France)—810 nm (M810L5, bandwidth 30 nm), 880 nm (M880D2 or M880L3, bandwidth 50 nm), 940 nm (M940L3, bandwidth 37 nm), 970 nm (M970L4, bandwidth 60 nm), or 1050 nm (M1050L4, bandwidth 37 nm)—and aspheric condenser lens ACL25416U or LC1054 were mounted to illuminate three cuvettes in Turret 4, while the fourth cuvette served as a dark control for comparison (Fig. S1). At defined time intervals, the absorption spectra of all four cuvettes were sequentially measured. The power at the cuvette positions was measured using a Thorlabs power meter (PM100D) equipped with an S130C slim sensor.

### Fluorescent labeling

Chromosomal DNA of F-J1 cell was stained with 2 μg/ml 4′,6-diamidino-2-phenylindole (DAPI) for 5 min at room temperature. For cell cytoplasmic membrane visualization, F-J1 cells were stained with 1.25 μg/ml FM4-64 (Invitrogen) at 0°C for 10 min and immediately subjected to fluorescence microscopy. For vesicle labeling, cells were incubated with FM4-64 at 30°C for 30 min prior to fluorescence imaging analysis. For gentamicin-fluorescein isothiocyanate (FITC) uptake analysis, F-J1 cells were cultured in medium as indicated containing 10 μg/ml gentamicin-FITC until reaching stationary phase. Cells were then collected by centrifugation and washed three times with phosphate-buffered saline (PBS) buffer (pH 7.5). Cell suspensions were mounted on glass slides with coverslips and examined under fluorescence microscopy.

### Genome sequencing and analysis

F-J1 cells were mechanically disrupted and genomic DNA was extracted using the method described previously [14]. The whole genome of F-J1 was sequenced using DNBSEQ-G99 and CycloneSEQ WuTong02 platform. The genome assembly and annotation are described in SI.

### Quantitative proteomic analysis

F-J1 cells were harvested by centrifugation and disrupted using a French press in PBS buffer with protease inhibitors. Proteins were extracted and precipitated with trichloroacetic acid (TCA), followed by acetone washes. Proteins were separated by sodium dodecyl sulfate–polyacrylamide gel electrophoresis (SDS–PAGE), excised, reduced with dithiothreitol (DTT), alkylated with iodoacetamide, and digested overnight with trypsin at 37°C. Nano liquid chromatography–tandem mass spectrometry (nanoLC–MS/MS) analysis was performed on a Q Exactive Plus mass spectrometer with a 117-min gradient elution. Mass spectrometry (MS) resolution was 70 000 and MS/MS was 17 500, with higher-energy collisional dissociation (HCD) fragmentation of the top 10 most intense ions. Label-free quantification (LFQ) employed MaxQuant 2.5.0.0 with false discovery rate (FDR) set at 0.01. Perseus 1.6.15 was used for statistical analysis, including PCA, log2 transformation, and *t-*test with permutation-based FDR. Differential proteins were considered significant with fold change >1 and *P* < .001. Five biological replicates were analyzed per condition. The mass spectrometry proteomics data have been deposited to the ProteomeXchange Consortium via the PRIDE [15] partner repository with the dataset identifier PXD069040, more details in SI.

### Statistical analysis

Statistical analyses were conducted using one-way or two-way analysis of variance (ANOVA), as appropriate, followed by Tukey’s multiple comparisons test. To evaluate the interaction between temperature and wavelength, a 3 × 4 two-way ANOVA was performed. Both wavelength (*P* < .001, η^2^p = 0.89) and temperature (*P* < .001, η^2^p = 0.93) have significant main effects on growth rate, with a highly significant interaction (*P* < .001, η^2^p = 0.87). Since the interaction was significant, the omnibus main effects are not directly interpretable; instead, the effect of wavelength must be examined separately at each temperature level. Simple effects analyses decomposed the interaction by conducting one-way ANOVAs for wavelength at each of the four temperatures (Table S1). Unless otherwise stated, all experiments were performed with at least three independent biological replicates. Differences were considered statistically significant at *P* < .05. Significance levels are denoted as follows: ns, not significant (*P* > .05); ^*^, *P* < .05; ^**^, *P* < .01; ^***^, *P* < .001; ^****^, *P* < .0001.

## Results

### Isolation of *R. mucilaginosa* strain F-J1

**Figure 1:**
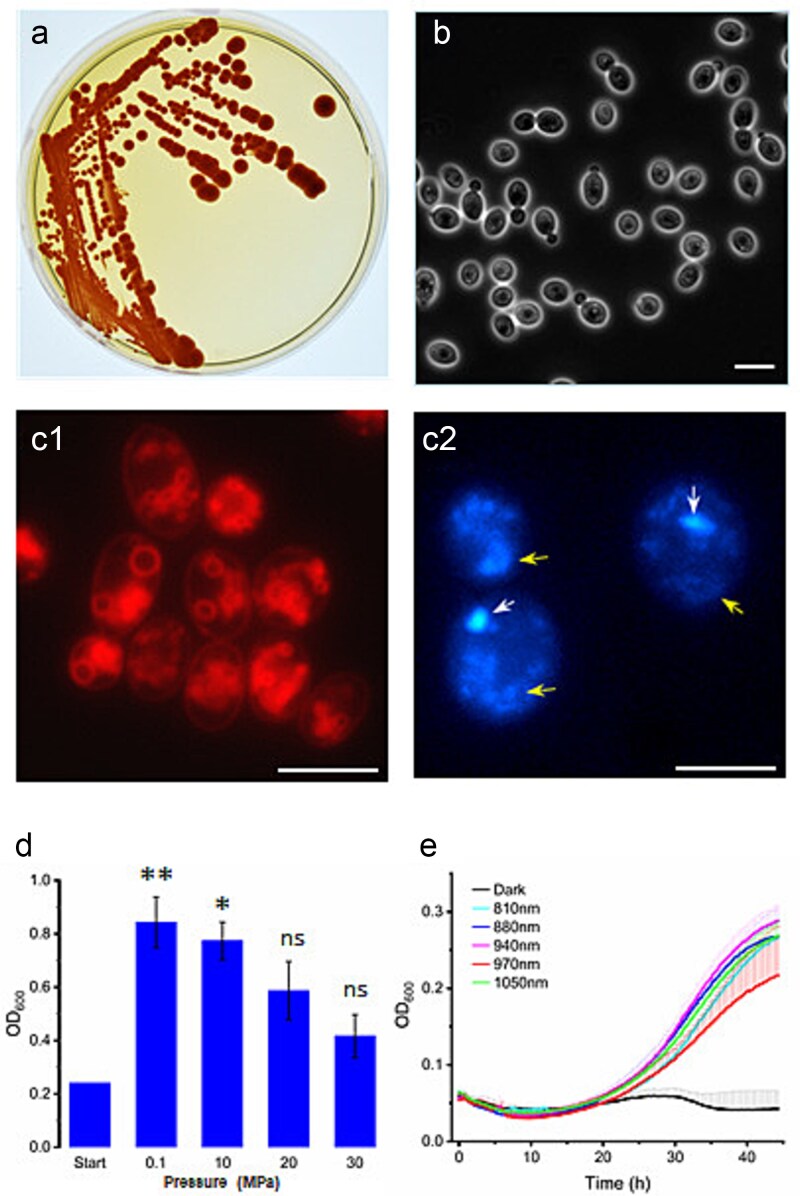
Morphology and growth characteristics of strain F-J1. (a) Colony and (b) cellular morphologies of F-J1. (c1) and (c2) show F-J1 cells stained with FM4-64 and DAPI, respectively. White and yellow arrows indicate potential nuclear and organelle chromosomes, respectively. Scale bar: 5 μm. (d) Final biomass yield of three independent (*n* = 3) F-J1 cultures after 22 h of incubation at 30°C under varying pressures. The symbols represent comparisons between each pressure condition and the start inoculation. ns, not significant (*P* > .05); ^*^, *P* < .05; ^**^, *P* < .01. (e) Growth of F-J1 exposed to NIR irradiation at the indicated wavelengths at 15°C. Values represent means from three independent experiments, with only positive standard deviations shown for clarity. Statistical analysis shows that all wavelength conditions were significantly different from the dark condition (*P* < .0001).

Among colonies cultured from IR-enriched samples, one notable colony exhibited a smooth, red appearance (Fig. 1a). Subsequent isolation yielded an axenic culture, with cells displaying budding yeast morphology under bright-field microscopy (Fig. 1b). FM4-64 membrane staining revealed vacuoles (Fig. 1c1, red circles), a eukaryotic cell hallmark. DAPI staining showed strong signals, likely nuclear chromosomes, and weaker signals potentially from organelle chromosomes (Fig. 1c2). Amplification and sequencing of the eukaryotic 18S rRNA gene revealed 99.94% identity with *R. mucilaginosa* strain LBMH1012, confirming its classification as a yeast. This strain was consequently designated *R. mucilaginosa* F-J1 and will be primarily referred to as F-J1 hereafter.

We sequenced the mitochondrial DNA (mtDNA) of F-J1, which was 41 408 bp in length with a G + C content of 40.5% (Fig. S2). This mtDNA exhibited 99.98% and 99.45% sequence identities (with 100% coverage) to the mtDNA of *R. mucilaginosa* strain GDMCC2.30 and *R. mucilaginosa* strain RIT389, respectively. The strain RIT389 mtDNA was ~6 kb larger and contained six additional ORFs compared to those of *R. mucilaginosa* strains F-J1 and GDMCC2.30 (Fig. S2). F-J1 mtDNA encoded 25 tRNAs, covering all 20 amino acids with three copies for *trnM*, and two copies each for *trnL, trnS*, and *trnV* (Fig. S2). This genetic architecture suggests independence from nuclear DNA (nuDNA)–encoded tRNAs and potentially less mtDNA transfer to nuDNA or mtDNA deletion during F-J1 mitochondrial evolution. Twenty-six genes encoded ribosomal rRNA (2), ribosomal binding protein (rps3), components of complexes I (6), III (1), and IV (3), and ATPase subunits (3)—all essential for energy production via oxidative phosphorylation—as well as 10 open reading frames (ORFs). Most ORFs were predicted as GIY-YIG endonucleases, primarily located in introns of various genes (Fig. S2), consistent with reports of GIY-YIG ORFs serving as mobile elements in mitochondrial introns during evolution [16].

F-J1 grew optimally under atmospheric pressure (0.1 MPa), while elevated hydrostatic pressure progressively inhibited growth (Fig. 1d), indicating a piezo-tolerant phenotype. Near-infrared (NIR) irradiation within the wavelength range of 810–1050 nm stimulated the growth of F-J1 compared to growth in darkness, further confirming the IR-enhanced growth characteristic of this strain (Fig. 1e). Increasing the light power of 880 nm enhanced this beneficial effect, reaching a plateau between 1.2 and 1.5 mW, followed by a slight decrease with further power augmentation. Unless otherwise specified, we subsequently used a power of 1.5 mW, corresponding to a power density (irradiance) of 3.1 mW/cm^2^ for all wavelengths used.

### Effect of temperature and near-infrared irradiation on the growth rate of F-J1

**Figure 2:**
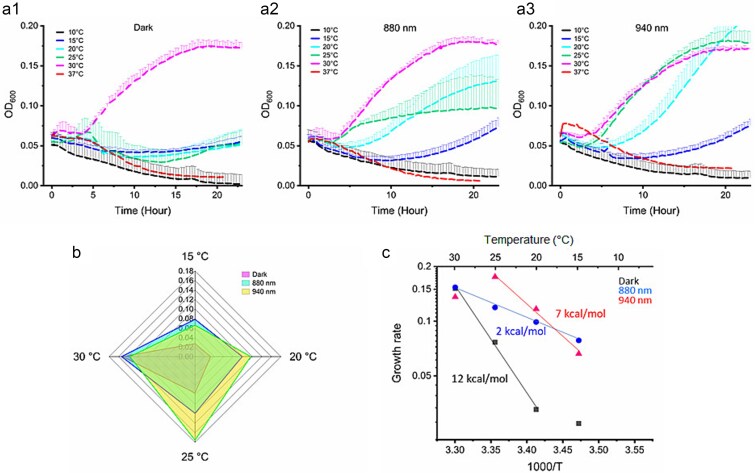
Temperature and NIR effects on F-J1 growth. Growth curves under darkness (a1), 880 nm (a2), and 940 nm (a3) NIR at various temperatures. Data are means of three experiments except for the cultures at 37°C where F-J1 didn’t grow even after 72 h; only positive SDs shown. (b) Radar plot of growth rates by temperature: magenta (dark), cyan (880 nm), and yellow (940 nm); border colors use additive mixing (red, green, blue). Statistical analysis shows both wavelength and temperature have significant main effects on growth rate. Statistical analysis is shown in Table S1. (c) Arrhenius plot of growth rates with slope and activation energy values.

We further examined the effect of NIR irradiation on the temperature-dependent growth of *R. mucilaginosa* F-J1. To simulate *in situ* sediment conditions of the sampling site, cultures were incubated without stirring, in the dark, at 10°C. Under these conditions, absorbance at 600 nm decreased due to cell sedimentation, and no significant growth was observed until 25 h postincubation (Fig. 2a1). The growth enhanced with increasing temperature from 10°C to 15°C, 20°C, and 25°C. Growth peaked at 30°C but was strongly inhibited at 37°C (Fig. 2a1). Similar to cultures in darkness, while no growth was noticed at 10°C and 37°C, evident growth of F-J1 was observed at 15°C, 20°C, and 25°C with exposure to IR irradiation at 880 and 940 nm, the wavelengths originally used in enhancing samples and isolation of F-J1 strain. Notably, cultures exposed to 880 nm NIR displayed large growth fluctuations at 20°C and 25°C (Fig. 2a2), whereas 940 nm NIR irradiation maximally stimulated growth at 25°C (Fig. 2a3).

The growth rate clearly demonstrated the influence of temperature and NIR irradiation on the growth of F-J1. Under dark conditions, the growth rate increased progressively with rising temperature, reaching its maximum at 30°C (Fig. 2b). NIR irradiation at both 880 and 940 nm further enhanced the growth rates at 15°C, 20°C, and 25°C. However, at 30°C, the maximum growth rates under NIR irradiation were similar to that observed in the dark.

The temperature sensitivity of bacterial growth can be analyzed using the Arrhenius law [17], which describes how reaction rates—in this case, growth rates—increase with temperature. Activation energy, calculated from the slope of the linear region in an Arrhenius plot (logarithm of growth rate versus the inverse of absolute temperature), reflects how sensitive the growth rate is to temperature changes. A higher activation energy, corresponding to a steeper Arrhenius slope, indicates greater sensitivity to temperature changes, even though all growth rates inherently depend on temperature. The activation energy of F-J1 grown in darkness was calculated to be 12 kcal/mol within the temperature range of 20°C–30°C (Fig. 2c), a value comparable to that of the budding yeast *S. cerevisiae* (11 kcal/mol) [18] and same to the value of 12 kcal/mol found in other yeast species [17]. In contrast, the activation energy of F-J1 decreased to 2 kcal/mol between 15°C and 30°C for cultures exposed to 880 nm NIR, and to 7 kcal/mol between 15°C and 25°C for those exposed to 940 nm NIR (Fig. 2c). The near-complete flattening of the Arrhenius slope under 880 nm NIR suggests that NIR irradiation supplies energy in a manner that bypasses the primary thermal rate-limiting step of growth.

### Near-infrared-mediated growth is independent of exogenous glucose supplementation

**Figure 3:**
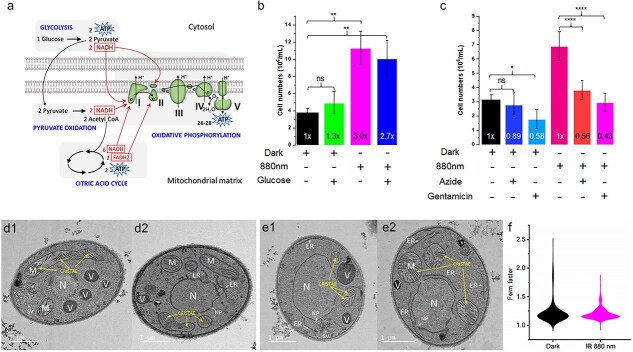
Mitochondrial metabolism involves in IR-stimulated F-J1 growth. (a) Schematic presentation of main energy production systems in yeast cells. (b) F-J1 biomass after 22-h cultivation at 20°C in YPS medium: without glucose in dark (black, 1×), supplemented with 0.1% glucose (green), irradiated by 880 nm NIR (magenta), or both glucose and NIR (blue). (c) F-J1 biomass after 24 h cultivation: in dark (black, 1×), and supplemented with 0.1 mM sodium azide (blue) or 10 μg/ml gentamicin in dark (light blue); or under 880 nm irradiation (red, 1×), and with azide (orange) or gentamicin (magenta). Numbers indicate fold-change relative to references (1×). Data: mean ± standard error with three independent replications. TEM images of F-J1 cells incubated in dark (d) or with 880 nm IR irradiation (e) at 20°C. Nuclear (N), nuclear pore complexes (NP), mitochondria (M), vacuoles (V), endoplasmic reticulum (ER), and secretory vesicles (SV) are indicated. (f) shows form factor (Perimeter^2^/(4π × Area)) of mitochondria in cells incubated in dark or exposed to IR, calculated from 207 and 206 mitochondria, respectively. For the statistical analysis, using Tukey’s multiple comparisons test.

Yeast typically derives adenosine triphosphate (ATP) for growth through glucose metabolism, which includes both cytoplasmic glycolysis and mitochondrial oxidative phosphorylation (OXPHOS) (Fig. 3a) [19]. To assess whether NIR-stimulated growth depends on exogenous chemical energy input, we used a carbon-depleted yeast extract-peptone medium lacking glucose and starch (YPS). Under these nutrient-limited conditions, glucose supplementation resulted in only a marginal, nonsignificant 1.3-fold increase in final biomass (Fig. 3b). In striking contrast, 880 nm NIR irradiation alone, in the absence of glucose supplementation, induced a robust and statistically significant three-fold increase in biomass production (*P* < .0018). Furthermore, combining glucose supplementation with NIR irradiation did not produce any additive or synergistic effect on growth. These results indicate that, under severe carbon limitation, NIR irradiation can stimulate a level of biomass production that far exceeds that supported by exogenous glucose alone. This suggests that NIR enhances the efficiency of endogenous carbon utilization or mitochondrial energy conversion, rather than merely supplementing external chemical energy.

### Mitochondrial metabolism underlies infrared-stimulated growth

We next examined the involvement of mitochondrial metabolism in NIR-stimulated growth. In yeast cells, the main power generators are the OXPHOS complexes I, III, IV, and ATP synthase, which are encoded by the mitochondrial genome and synthesized by mitochondrial ribosomes (Fig. 3a). Consequently, inhibiting OXPHOS activity or interrupting mitochondrial protein synthesis severely impairs mitochondrial energy metabolism.

Azide is a well-established inhibitor of cytochrome c oxidase. By binding to the heme iron of the enzyme, it blocks electron transfer to oxygen and halts aerobic respiration [20]. This inhibition of the electron transport chain reduces ATP production and thereby impairs cell growth. We found that 1 mM azide completely abolished F-J1 growth, demonstrating the strain’s high sensitivity to this inhibitor. At 0.1 mM, azide reduced final biomass by 11% in dark cultures, a change that was statistically nonsignificant. In contrast, under 880 nm NIR irradiation, the same concentration significantly reduced biomass by 44% (*P* < .0001) (Fig. 3c). This differential sensitivity indicates that NIR-stimulated growth has a greater absolute requirement for OXPHOS activity than basal growth in darkness.

An antibiotic sensitivity assay showed that *R. mucilaginosa* strain F-J1 was sensitive to aminoglycoside antibiotics—gentamicin (Gm), kanamycin, and hygromycin—while remaining resistant to other tested antibiotics (Fig. S3A). Aminoglycoside antibiotics function by interacting with specific nucleotides in the A-site (aminoacyl–tRNA binding site) of the bacterial 16S rRNA. This binding occurs within helix 44 of the decoding center (Fig. S3B), disrupting the ribosome’s ability to accurately read mRNA. The resulting synthesis of defective proteins leads to cellular damage and, ultimately, cell death.

We sequenced the genomes of *R. mucilaginosa* F-J1 and found that the nucleotide target sites for Gm are conserved in the mitochondrial small subunit (Mito-SSU) rRNA but absent in the cytoplasmic small subunit (Cyto-SSU) rRNA (Fig. S3B). This conservation of Gm binding sites likely accounts for the sensitivity of F-J1 to these antibiotics. As shown in Fig. 3c, 10 μg/ml Gm reduced final biomass by 42% in dark cultures (*P* < .05). In cultures exposed to 880-nm NIR light, however, the reduction was even greater at 57% (*P* < .0001). The enhanced inhibitory effect under NIR irradiation confirms that the stimulation of F-J1 growth by NIR depends on mitochondrial ribosome activity.

### Mitochondrial ultrastructure is modulated by near-infrared irradiation in F-J1

To investigate the mechanisms behind NIR-stimulated growth, we used transmission electron microscopy (TEM) to analyze its effects on cellular structures. TEM imaging of F-J1 cells revealed typical eukaryotic organization, including a nuclear envelope with nuclear pore complexes, electron-dense vacuoles, endoplasmic reticulum, secretory vesicles, and mitochondria (Fig. 3d and e). Given the close relationship between mitochondrial structure and metabolic function, we focused on mitochondrial morphology. Notably, dark-grown cells exhibited diverse mitochondrial morphologies, including not only the typical oval shape but also elongated and irregular forms (Fig. 3d). In contrast, NIR-irradiated cells predominantly contained mitochondria with a more uniform oval morphology—suggesting increased fragmentation (Fig. 3e). Such fragmentation is often associated with enhanced metabolic activity and mitochondrial fission, processes critical for efficient mitochondrial distribution and function [21, 22].

The mitochondrial form factor (a measure of circularity) was >1 under both conditions, confirming an overall oval shape (Fig. 3f). However, dark-grown cells had a higher form factor, consistent with their more elongated or irregular morphology compared to NIR-exposed cells. Mitochondrial hypertrophy, characterized by increased volume, typically reflects an adaptive response to elevated energy demands. Notably, NIR irradiation promoted mitochondria with a more regular oval shape, indicative of enhanced respiratory capacity—consistent with the observed NIR-stimulated growth.

### Effect of near-infrared irradiation on protein synthesis

Proteomic analysis of five independent replicates identified a core set of 2425 proteins consistently expressed in both IR-exposed and dark-grown cultures. Within this set, 5 proteins showed significant upregulation, and 22 were significantly downregulated in the IR-irradiated group compared to the dark-grown control group (Table S2, Fig. S4). The most notably upregulated protein, with a 6.7-fold increase in abundance, was mitochondrial ribosomal protein S18 (MRPS18). Other upregulated proteins included a ubiquitin carboxyl-terminal hydrolase, a molecular chaperone, and the regulatory subunit of the dimeric UBA3-ULA1 E1 enzyme. Collectively, these proteins primarily participate in protein synthesis and turnover. Conversely, the downregulated proteins exhibited a broader range of functions, lacking a specific association with energy metabolism or anabolic processes.

**Figure 4:**
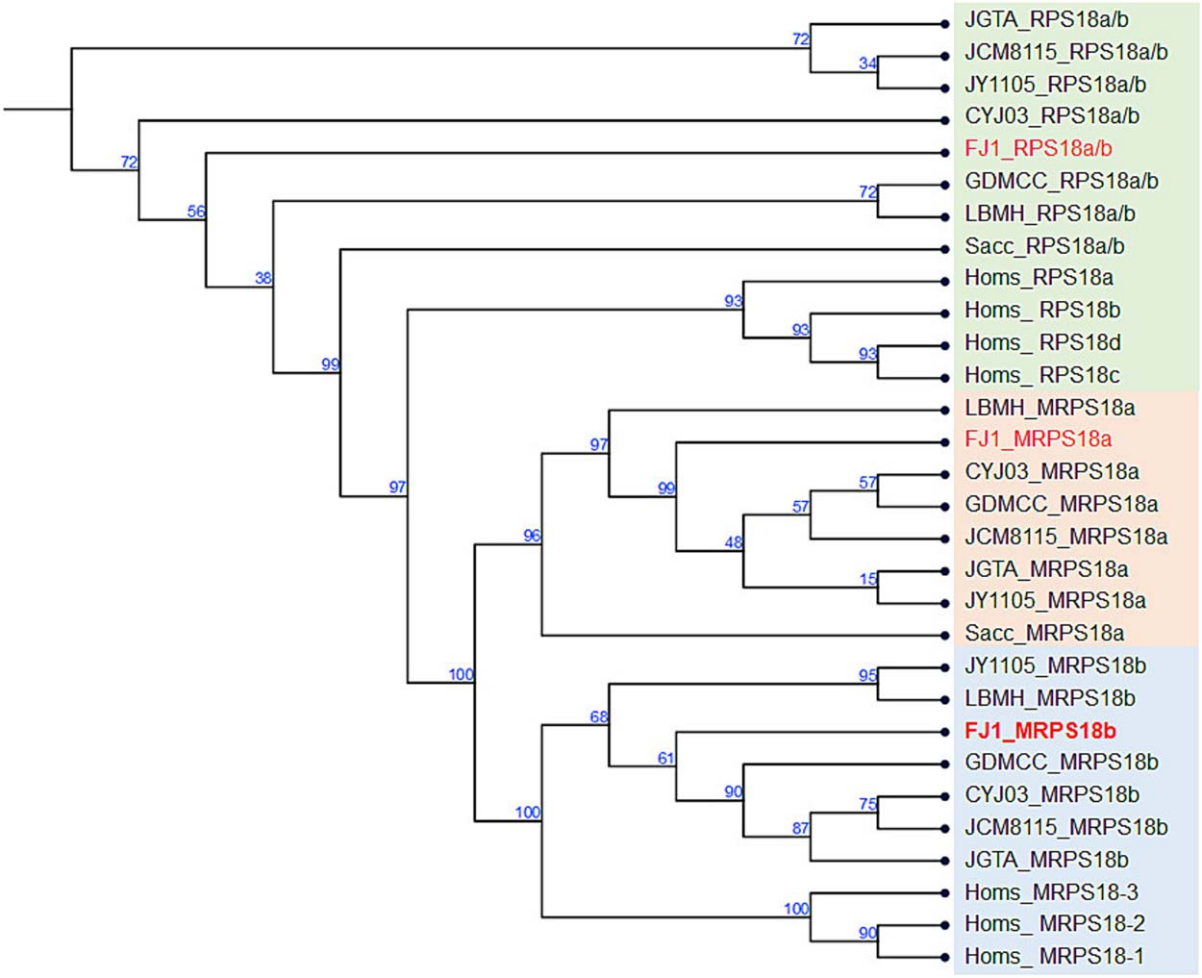
Phylogenetic analysis of the ribosomal protein S18. Using the two mitochondrial protein S18 (FJ1_MRPS18a and FJ1_MRPS18b, in red) and the cytoplasmic ribosomal protein S18 (FJ1_RPS18a/b, red) of F-J1 as queries, their counterparts were identified in the genomes of six additional *R. mucilaginosa* strains, in *S. cerevisiae* and *Homo sapiens*. The sequence identity and the method used are indicated in SI. The light-green, orange, and blue colors highlight the cytoplasmic RPS18 and the two groups of mitochondrial MRPS18, respectively.

MRPS18 is an essential component of the mitochondrial small ribosomal subunit required for ribosome assembly and mitochondrial translation [23]. Across eukaryotes, MRPS18 has undergone lineage-specific duplication and loss. Metazoans, including humans, retain three paralogs (S18-1, S18-2, and S18-3) [24], whereas *Saccharomyces cerevisiae* harbors a single S18 copy clustering with protist homologs, consistent with ancient duplication followed by differential retention. Our phylogenomic analysis confirmed a single S18 in *S. cerevisiae* but identified two distinct paralogs in all seven examined strains of *R. mucilaginosa*. FJ1_MRPS18a grouped with the S18 of *S. cerevisiae*, whereas the NIR-responsive FJ1_MRPS18b clustered with the three human isoforms (Fig. 4; Table S3), suggesting functional divergence.

Consistently, two additional *R. mucilaginosa* strains (DSM 13621 and DSM 18184) showed growth stimulation under 880 and 940 nm NIR irradiation, whereas *S. cerevisiae* exhibited no response (Fig. S5). This species-specific phenotype correlates with the presence of MRPS18b orthologs. To test causality, we heterologously expressed *R. mucilaginosa* F J1 MRPS18b in nonresponsive *S. cerevisiae*. Remarkably, this single gene transfer was sufficient to confer NIR stimulated growth (Fig. S5), identifying MRPS18b as a lineage specific, gain-of-function mitochondrial determinant of NIR responsiveness. The downstream mechanisms by which MRPS18b enables NIR-enhanced metabolism remain to be elucidated.

## Discussion

The prevailing view of hydrothermal vent ecology holds that chemosynthesis is the primary energy source sustaining deep-sea life, with food webs ultimately dependent on geochemical energy [1]. Our findings expand this framework by demonstrating that geothermal NIR can directly stimulate the growth of a deep-sea eukaryotic microbe.

The most robust conclusion of this study is that NIR irradiation enhances growth of *R. mucilaginosa* F-J1 through a mitochondria-centered metabolic pathway. Multiple independent lines of evidence support this conclusion. First, NIR exposure significantly increased growth rates across multiple temperatures and reduced the apparent activation energy for growth, indicating a strong metabolic effect that cannot be explained solely by conventional temperature dependence. Second, pharmacological inhibition experiments demonstrated that this response depends on mitochondrial oxidative phosphorylation and mitochondrial ribosomal activity, directly linking the phenotype to mitochondrial function. Third, transmission electron microscopy revealed NIR-associated remodeling of mitochondrial morphology consistent with elevated respiratory activity. Fourth, proteomic analysis identified MRPS18b as the most strongly upregulated protein under NIR exposure. Importantly, heterologous expression of MRPS18b in *S. cerevisiae* was sufficient to confer NIR responsiveness, establishing this mitochondrial ribosomal protein as a lineage-specific determinant of the phenotype. Together, these findings provide strong empirical evidence that geothermal NIR radiation acts as a biologically relevant environmental stimulus capable of enhancing mitochondrial metabolism in deep-sea yeast.

Importantly, the proximal biophysical mechanism by which NIR exposure is initially sensed remains unresolved. Rather than proposing a definitive physical mechanism, we consider this an important open question for future work. Potential mechanisms could operate in parallel or sequentially, including NIR photoperception and signaling cascades, direct absorption by mitochondrial chromophores that could contribute to cellular energy capture, indirect effects via NIR-induced changes in membrane fluidity, or photothermal micro-heating at the organellar scale that is not captured by bulk temperature measurements. Direct investigation of mitochondrial membrane potential, ATP production, redox state, and potential photophysical or signaling intermediates will be essential to define how NIR exposure is transduced into enhanced metabolism.

By focusing on the biological response itself, our study reveals a previously unrecognized capacity of deep-sea eukaryotic microbes to utilize geothermal radiative energy as a growth-promoting input. This expands the energetic framework of hydrothermal vent ecosystems beyond the traditional dichotomy of chemosynthesis and photosynthesis.

## Supplementary Material

F-J1_SI_ISME_Comm_20260510_ycag129

## Data Availability

All data generated or analyzed during this study are included in this published article and its supplementary information files. The complete genome sequence of *R. mucilaginosa* F-J1 was deposited in the NCBI (https://www.ncbi.nlm.nih.gov/) under the accession number PRJNA1326177 (BioProject) and SAMN51257182 (BioSample). The mitochondrial genome sequence has been deposited in the GenBank database with accession number PV917115. The mass spectrometry proteomics data are available via ProteomeXchange with identifier PXD069040.

## References

[1] Dick GJ . The microbiomes of deep-sea hydrothermal vents: distributed globally, shaped locally. *Nat Rev Microbiol* 2019;17:271–83. 30867583 10.1038/s41579-019-0160-2

[2] Van Dover CL, Szuts EZ, Chamberlain SC et al. A novel eye in 'eyeless' shrimp from hydrothermal vents of the mid-Atlantic ridge. *Nature* 1989;337:458–60. 10.1038/337458a0 2915690

[3] Van Dover CL, Reynolds GT, Chave AD et al. Light at deep-sea hydrothermal vents. *Geophys Res Lett* 1996;23:2049–52. 10.1029/96GL02151

[4] Yurkov V, Beatty JT. Isolation of aerobic anoxygenic photosynthetic bacteria from black smoker plume waters of the juan de fuca ridge in the pacific ocean. *Appl Environ Microbiol* 1998;64:337–41. 10.1128/AEM.64.1.337-341.1998 16349490 PMC124714

[5] Beatty JT, Overmann J, Lince MT et al. An obligately photosynthetic bacterial anaerobe from a deep-sea hydrothermal vent. *Proc Natl Acad Sci USA* 2005;102:9306–10. 10.1073/pnas.0503674102 15967984 PMC1166624

[6] Li Y, Zhu J, Li Q et al. Non-linear frequency-doubling up-conversion in sulfide minerals enables deep-sea oxygenic photosynthesis. *Natl Sci Rev* 2025;12:nwaf219. 10.1093/nsr/nwaf219 40584012 PMC12202869

[7] Nisbet EG, Cann JR, Van Dover CL. Origins of photosynthesis. *Nature* 1995;373:479–80. 10.1038/373479a0

[8] Martin W, Baross J, Kelley D et al. Hydrothermal vents and the origin of life. *Nat Rev Microbiol* 2008;6:805–14. 10.1038/nrmicro1991 18820700

[9] Chen H, Li DH, Jiang AJ et al. Metagenomic analysis reveals wide distribution of phototrophic bacteria in hydrothermal vents on the ultraslow-spreading southwest indian ridge. *Mar Life Sci Technol* 2022;4:255–67. 10.1007/s42995-021-00121-y 37073225 PMC10077154

[10] Dai J, Li XG, Zhang TY et al. Illuminating a bacterial adaptation mechanism: infrared-driven cell division in deep-sea hydrothermal vent environments. *Innov Geosci* 2024;2:100050. 10.59717/j.xinn-geo.2024.100050

[11] Dai J, Li XG, Zhang WJ et al. Tepidibacter hydrothermalis sp. Nov., a novel anaerobic bacterium isolated from a deep-sea hydrothermal vent. *Int J Syst Evol Microbiol* 2023;73:006151. 10.1099/ijsem.0.006151 37921840

[12] Zhao R . Isolation and Identification of Photoenergetic Microorganisms in Deep-Sea Hydrothermal Vents and the Adaptation Mechanism. Master's thesis. Beijing, China: University of Chinese Academy of Sciences, 2023.

[13] Ghaddar N, Corda Y, Luciano P et al. The compass subunit spp1 protects nascent DNA at the tus/ter replication fork barrier by limiting DNA availability to nucleases. *Nat Commun* 2023;14:5430. 10.1038/s41467-023-41100-4 37669924 PMC10480214

[14] Wang Y, Zhou J, Zou Y et al. Fungal commensalism modulated by a dual-action phosphate transceptor. *Cell Rep* 2022;38:110293. 10.1016/j.celrep.2021.110293 35081357

[15] Perez-Riverol Y, Bandla C, Kundu DJ et al. The pride database at 20 years: 2025 update. *Nucleic Acids Res* 2025;53:D543–53. 10.1093/nar/gkae1011 39494541 PMC11701690

[16] Saguez C, Lecellier G, Koll F. Intronic giy-yig endonuclease gene in the mitochondrial genome of podospora curvicolla: evidence for mobility. *Nucleic Acids Res* 2000;28:1299–306. 10.1093/nar/28.6.1299 10684923 PMC111034

[17] Knapp BD, Huang KC. The effects of temperature on cellular physiology. *Annu Rev Biophys* 2022;51:499–526. 10.1146/annurev-biophys-112221-074832 35534014

[18] Vanoni M, Vai M, Frascotti G. Effects of temperature on the yeast cell cycle analyzed by flow cytometry. *Cytometry* 1984;5:530–3. 10.1002/cyto.990050515 6386390

[19] Jiang M, Wang L, Sheng H. Mitochondria in depression: the dysfunction of mitochondrial energy metabolism and quality control systems. *CNS Neurosci Ther* 2024;30:e14576. 10.1111/cns.14576 38334212 PMC10853899

[20] Guerin MN, Ellis TS, Ware MJ et al. Evolution of a biological thermocouple by adaptation of cytochrome c oxidase in a subterrestrial metazoan, halicephalobus mephisto. *Commun Biol* 2024;7:1214. 10.1038/s42003-024-06886-z 39342021 PMC11439043

[21] Okamoto K, Shaw JM. Mitochondrial morphology and dynamics in yeast and multicellular eukaryotes. *Annu Rev Genet* 2005;39:503–36. 10.1146/annurev.genet.38.072902.093019 16285870

[22] Westermann B . Mitochondrial dynamics in model organisms: what yeasts, worms and flies have taught us about fusion and fission of mitochondria. *Semin Cell Dev Biol* 2010;21:542–9. 10.1016/j.semcdb.2009.12.003 20006727

[23] Desai N, Brown A, Amunts A et al. The structure of the yeast mitochondrial ribosome. *Science* 2017;355:528–31. 10.1126/science.aal2415 28154081 PMC5295643

[24] Mushtaq M, Ali RH, Kashuba V et al. S18 family of mitochondrial ribosomal proteins: evolutionary history and gly132 polymorphism in colon carcinoma. *Oncotarget* 2016;7:55649–62. 10.18632/oncotarget.10957 27489352 PMC5342443

