# Supplementary material for: Infrared stimulation of mitochondrial metabolism in deep-sea yeast expands the energetic framework of hydrothermal ecosystems
Source: ISME Commun. 2026 May 19;6(1):ycag129. doi: 10.1093/ismeco/ycag129 (PMC13440299; doi:10.1093/ismeco/ycag129)
Supplement: Supplementary file 1 [file f-j1_si_isme_comm_20260510_ycag129.docx]

**Supporting Information for**

**Infrared stimulation of mitochondrial metabolism in deep-sea yeast expands the energetic framework of hydrothermal ecosystems**

Jie Dai^1,2^, Luyao Zhang^3^, Yingshan Li^4^, Rui Zhao^5^, Xue-Gong Li^5,6,7^, Jihong Liu^3^, Artemis Kosta^8^, Hugo Le Guenno^8^, Salomé Sauvage^9^, Wei-Jia Zhang^5,6.7^, Régine Lebrun^9^, Yue Shen^4,10^, Huanming Yang^10^, Changbin Chen^3^, Long-Fei Wu^1,^*

^1^Aix Marseille University, CNRS, LCB, IMM, IM2B, CENTURI; Marseille, 13402, France.

^2^Research Centre of Ecology & Environment for Coastal Area and Deep Sea, Southern Marine Science and Engineering Guangdong Laboratory (Guangzhou), Guangzhou 511458, China

^3^The Joint Laboratory for Biomedical Research and Pharmaceutical Innovation, Unit of Pathogenic Fungal Infection & Host Immunity, Shanghai Institute of Materia Medica, Chinese Academy of Sciences, Shanghai, China.

^4^BGI Research; Changzhou, 213100, China.

^5^Laboratory of Deep-Sea Microbial Cell Biology, Institute of Deep-sea Science and Engineering, Chinese Academy of Sciences; Sanya, 572000, China.

^6^CAS Key Laboratory for Experimental Study under Deep-sea Extreme Conditions, Institute of Deep-sea Science and Engineering, Chinese Academy of Sciences; Sanya, 572000, China.

^7^Institution of Deep-sea Life Sciences, IDSSE-BGI, Hainan Deep-sea Technology Laboratory; Sanya, 572000, China.

^8^Microscopy Core Facility, FR3479 IMM, CNRS, Aix Marseille University; Marseille, 13000, France.

^9^Marseille Protéomique, IMM, CNRS, Aix Marseille University; Marseille, 13000,France.

^10^Guangdong Provincial Key Laboratory of Genome Read and Write, BGI Research; Shenzhen, 518083, China.

* Corresponding author: Long-Fei WU, Laboratoire de Chimie Bacterienne, UMR7283 CNRS-AMU, 31 chemin Joseph Aiguier, 13009 Marseille, France. Phone 33-4 9116 4157 ; email : [wu@imm.cnrs.fr](mailto:wu@imm.cnrs.fr)

**This PDF file includes:**

Supporting text

Figures S1 to S5

Tables S1 to S2

SI References

Supporting Information Text

Optical microscopy

Routine optical microscopy observation was performed with Zeiss Axiostar Plus, Zeiss Axio Vert 200M, and Olympus BX51. Fluorescent imaging was performed with a Nikon TiE microscope. To perform laser confocal analysis, cells were observed with the Olympus FV1000 microscope with laser 405 nm excitation and 425–475 nm emission for DAPI and 543 nm excitation and 555–655 nm emission for FM4-64 as described previously (1, 2).

Transmission electron microscopy

Yeast cells were pelleted, fixed, and processed following the protocol described by Write et al. (3). Specifically, cells were fixed in 2% glutaraldehyde prepared in HEPES (4-(2-hydroxyethyl)-1-piperazineethanesulfonic acid) buffer, then washed twice with the same buffer. They were subsequently transferred to a 2% aqueous solution of potassium permanganate for 45 minutes. After treatment, the cells were thoroughly washed 6–7 times with double-distilled water (ddH₂O), followed by dehydration through a graded ethanol series (25%, 50%, 75%, and 100%). The pellets were then transferred to fresh vials and resuspended in a 2:1 mixture of ethanol and Spurr’s resin. After a 2-hour incubation, the mixture was replaced with a 1:1 ethanol: resin solution and incubated overnight. This was followed by two changes of 100% resin, each for 2 hours. Fragments of the pellets were placed in BEEM capsules and polymerized at 60°C for 48 hours. Ultrathin sections (60–90 nm) were cut using an ultracryomicrotome (EM UC7, Leica) and stained with uranyl acetate and lead citrate, as described by Reynolds (4). Samples were imaged with a Tecnai 200 kV electron microscope (FEI), and digital images were acquired using a OneView CMOS camera (Gatan).

Cultivation under high hydrostatic pressure

For high-pressure incubation, the preculture was inoculated into GYPS medium. A volume of 2 mL of the liquid culture and 2 mL of air were drawn into a 5 mL syringe, which was subsequently placed in a water-filled high-pressure vessel. Hydrostatic pressure was applied manually using a hand-operated pump. The vessels were incubated overnight at 30 °C. After incubation, the cultures were retrieved from the syringes and transferred into cuvettes. Optical density at 600 nm was determined using a Cary-50 spectrophotometer (Agilent, France)

Cultivation with antibiotics

Antibiotic-containing agar plates were prepared by adding the sterile antibiotic solution to the autoclaved agar medium cooled to approximately 50–55 °C. The medium was mixed thoroughly and poured into sterile Petri dishes under aseptic conditions. Plates were allowed to solidify at room temperature. Yeast cells were then plated onto plate and incubation at 30 °C for two days. Antibiotics were used at the following working concentrations: ampicillin (50 µg/mL), chloramphenicol (20 µg/mL), kanamycin (50 µg/mL), gentamicin (15 µg/mL), and hygromycin (170 µg/mL).

Image Analysis

Mitochondrial segmentation was performed using Empanada (5) with the pre-trained MitoNet_v1_mini model and visualized using napari. Images (initial size: 4096 × 4096 pixels) were down sampled by a factor of 4 (final size: 1024 × 1024) prior to segmentation. The segmentation was run with the following parameters: Segmentation Confidence Threshold = 0.10, Center Confidence Threshold = 0.05, and Minimum Distance Between Centers = 3. Segmentations were visually inspected in napari, and manual corrections were performed using Fiji (ImageJ) to refine mitochondrial contours where necessary. The resulting binary masks were then analyzed to extract mitochondrial form factor and other morphological features.

Genomic analysis

For DNASEQ-G99 sequencing, the genomic DNA was first fragmented using the MGIEasy Fast PCR-FREE FS Library Prep Set. Sequencing adapters were then added to the DNA library using the MGIEasy UDB Primers Adapter Kit A. A DNB library was prepared, and paired-end sequencing (2X150 bp) was conducted, yielding 2.2G raw sequence data (in fasta format) for each end. The CycloneSEQ library was prepared following the manufacturer’s guidelines. The libraries were quantified by Qubit fluorometer and sent for sequencing on the CycloneSEQ WuTong02 platform. Quality control of the MGI raw reads was performed using fastp (v0.23.4) (6) with default parameters. The filtered reads were assembled using Spades (v3.15.5) (7). To improve assembly accuracy, RagTag v2.1.0 (8) was applied for reference-guided correction. The RagTag correct module was used to identify and correct misassemblies in the contigs by aligning them to a closely related reference genome (NCBI ID: GCA_016584205.1), thereby increasing the assembly's accuracy and reducing structural errors. Subsequently, scaffolding was performed using the RagTag scaffold module. The corrected contigs were reordered and oriented according to the reference, resulting in a more continuous and structured scaffold assembly. For gene prediction, an unsupervised training-based approach was employed, leveraging transcriptome evidence. Transcript sequences were aligned to the assembled genome, and gene models were predicted de novo using GeneMark-ET (9) based on the aligned transcripts. Functional annotation of predicted protein-coding genes was conducted using EggNOG-mapper v2 (10), which performs orthology-based annotation by aligning protein sequences against the EggNOG database.

The F-J1 mitochondria genome was distinguished from nuclear genome according to its high sequencing coverage. The coverage of mitochondria genome was 6048X, approximately 20 times deeper than nuclear genome contigs whose average coverage were between 30-40X. The F-J1 mitochondria genome was annotated using GeSeq web server (https://chlorobox.mpimp-golm.mpg.de/geseq.html) (11) and manually corrected in Snapgene v8.0.

Protein extraction and digestion

Yeast cells were harvested by centrifugation (7,000 rpm, 10 min). Pellets were then resuspended in PBS buffer (pH 7.4) supplemented with protease inhibitors (cOmplete, EDTA-free Protease inhibitor cocktail, Roche. France). Cells were disrupted using a French press at 2 MPa for three cycles. The resulting suspension was centrifuged (4 °C, 7,000 g, 30 min), and the supernatant, containing the crude protein extract, was collected. Proteins were precipitated by incubating the supernatant with two volumes of cold pure trichloroacetic acid (TCA) for 30 min at 4 °C. After centrifugation (14,000 rpm, 5 min), the supernatant was discarded, and the protein pellet was washed twice with 200 µL of cold acetone. Each wash was followed by centrifugation (14,000 rpm, 5 min) and supernatant removal. The final pellet was air-dried by placing the tube in a 95 °C heat block for 5 min to evaporate residual acetone.

Extracted proteins, 20 µg, were mixed with sodium dodecyl sulfate (SDS) loading buffer (62 mM Tris-HCl pH 6.8, 20 % glycerol (v/v), 0.04 % bromophenol blue (w/v), 0.1 M dithiothreitol (DTT), SDS 4 % (w/v)) then loaded onto a SDS-PAGE stacking gel 7 %. A short electrophoresis was performed (150V, 8 min) to concentrate proteins. After migration, gels were stained 20 min with ReadyBlueTM Protein Gel Stain (Sigma Aldrich) and destained with deionized water. The revealed protein bands were excised and washed three times during 10 min with 50 % ammonium bicarbonate 100 mM/50 % acetonitrile (ACN), before to be dried with 100 % ACN during 15 min. Disulfide bonds were disrupted with 10 mM DTT, 45 min at 56 °C in the dark; and cysteines were alkylated with 55 mM iodoacetamide, 30 min in the dark. Gel bands were then treated with 50 % ammonium bicarbonate 100 mM/50 % ACN (10 min, 2 times) and dried again with 100 % ACN during 15 min. Proteins were digested with Trypsin/LysC protease mix (Promega) with final 0.025 % ProteaseMax (v/v) overnight at 37 °C in ammonium bicarbonate 25 mM. Finally, peptides were extracted with 0.1 % trifluoroacetic acid (TFA) followed by 100 % ACN (5 min each, 2 times) and then dried. For each condition, five biological replicates were carried.

NanoLC-MS/MS protein analyses

Tryptic peptides were solubilized in H_2_O with 0.05 % TFA and 2 % ACN, and analyzed on a Q Exactive Plus (Thermo Scientific) mass spectrometer equipped with a nanoEasy Spray source coupled with a Vanquish Neo (Thermo Scientific) liquid chromatography. Peptides were injected onto an enrichment column (C18 Acclaim PepMap RSLC,75 µm × 500 mm, 2 µm, 100 Ǻ, Thermo Scientific). The buffers for the mobile phase were composed of 100 % H_2_O/0.1 % formic acid (FA) (A) and 80 % ACN/20 % H_2_O/0.1 % FA (B). The elution gradient duration was 117 min: 2 % to 25 % B from 0 to 90 min, 25 % to 50 % B from 90 to 110 min, and 50 % to 99 % B from 110 to 117 min. The flow rate was 0.3 µL/min. The temperature of the column was set at 40 °C. The maximum injection time was set to 100 ms. The capillary voltage was set to 1.9 kV. The temperature of the capillary was 280 °C. The Data dependent analysis was performed in HCD fragmentation mode of the 10 most intense ions selected (Top 10), with Nitrogen used as the collision gas. The m/z detection range was 350-1,900. The resolution was set to 70,000 for Full MS, and 17,500 for MS/MS fragmentation. All spectra obtained were exported in “.raw” format to identify peptides.

Quantitative proteomic analysis

Data were analysed by Maxquant (Max Planck Institute, Deutschland, https://www.maxquant.org, version 2.5.0.0) for label free quantification (LFQ), with global settings as local database of strain F-J1 (7,633 proteins) and in the group-specific parameters, the digestion was specific by trypsin, and amino acid modifications were chosen such carbamidomethylation on cysteine (fixed), oxidation on methionine (variable) and acetylation on protein N-term (variable). In LFQ experiment, all samples were combined in two groups: IR 880nm-cultured cells and dark-cultured cells. Peptides and proteins were identified by matching the recorded MS/MS data with in silico MS/MS data, two missed cleavages were allowed, the false discovery rate (FDR) value was selected as 0.01 for reliable identification and the option « match between runs » was selected. The option « iBAQ » for intensity-based absolute quantification was also generated and brought rough estimations of the relative abundance of the proteins within each sample.,. Then, the normalization type was classic, and the mass tolerance used was 20 and 4.5 ppm for the first and main search, respectively. The resulting « proteinGroups.txt » file was then used in Perseus software (12), where the proteins identified by site, matching to the reverse database and the classical contaminants were excluded. PCA was performed to show the data variance, before transforming the protein LFQ intensities according to log_2_ in order to facilitate the calculation of the protein expression fold change. A histogram was then built to confirm if the transformed matrix followed the normal distribution. Next, samples were grouped in categories according to their assays, filtered on three valid values in at least one group, and missing values were imputed assuming a normal distribution of 0.3 width and 1.8 downshifts. Finally, significant data points were determined using a t-test with a 250-permutation based FDR calculation (with 5 % FDR and S0 = 0.1) and represented as volcano plot. Differential proteins were considered significant if their fold change was >2 (difference in log_2_ (FC) > 1 on the x axis) with p-value < 0.1 (-log_10_(p-value) > 1 on the y axis).

Hetero-expression of F-J1 MRP18b in *S. serevisiae*

The coding sequence of MRP18b was synthesized after codon optimization for *S. cerevisiae* and cloned into the shuttle vector pRS416 under the control of the GAL1 promoter. The recombinant plasmid was transformed into *S. cerevisiae* BY4741 (MATa *his3Δ1 leu2Δ0 met15Δ0 ura3Δ0*) using a lithium acetate–based chemical method. Transformants, FJ10461, were selected on SC–URA plates (per 1L of ddH_2_O: Yeast nitrogen base 1.7 g, (NH_4_)_2_SO_4_ 5 g, Drop-out amino acid (-U) 1.47 g). Preculture were prepared by inoculation of FJ10461 in SC–URA plus 2% glucose. To express MRP18b, FJ120461 preculture was inoculated in SC–URA media with 2% galactose to induceMRP18b expression.

Fig. S1. Automated cultivation and spectral absorption analysis.

(A) The Turret 4 Temperature-Controlled Cuvette Holder from Quantum Northwest Inc. (www.qnw.com) was integrated into a Cary-60 spectrophotometer (Agilent, France). (B) Three Thorlabs LEDs (Thorlabs, France) were mounted for illumination. (C) At defined time intervals, the absorption spectra of the four cuvettes were sequentially measured.

Fig. S2. Structure and gene composition of mitochondrial DNA from three closely related *R. mucilaginosa* strains.

The figures are drawn using Snapgene v8.0 software. The mtDNA sequence of F-J1 has been deposited in the GenBank database with accession number PV917115. The original sequence data for *R. mucilaginosa* strains GDMCC2.30 and RIT389 were downloaded from Genbank websites https://www.ncbi.nlm.nih.gov/nuccore/CP137882.1 and https://www.ncbi.nlm.nih.gov/nuccore/NC_036340.1, respectively

Fig. S3. Antibiotic sensitivity of *R. mucilaginosa* F-J1.

(A) Tested on YPD plates with antibiotics as indicated. The strains used are bacterium *Escherichia coli* K12(top-left), yeast F-J1 (top-right) and *Candida albicans* SC5314 (bottom). (B) Aminoglycoside target site in ribosomal rRNA. Conserved residues (bold black) and non-conserved residues (blue) are depicted. Red highlights mark gentamicin-binding nucleotides within the decoding center. Italicized and underlined letters denote nucleotides not conserved between prokaryotic 16S and eukaryotic 18S rRNAs. The rRNA sequence were downloaded from GenBank (NR_024570.1; CP137882.1; NC_027264.1; AB021668.1; NG_063315.1;). The target information was compiled from previous studies (13).

Fig. S4. Protein abundance between IR 800 nm and dark.

Dots represent proteins whose abundance varied between the two conditions. Five biological replicates per condition were analyzed. Fold change was calculated as the ratio of IR 880 nm to dark. Blue dots: more abundant in dark culture; green dots: more abundant in IR 880 nm. Proteins with |log2(fold change)| > 1 on the x-axis and -log10(p-value) > 1 on the y-axis are considered as statistically significant (red dots with protein ID). The identified proteins are listed in the Table S2. FJ10461 corresponds to mitochondrial ribosome protein S18b.

Fig. S5. Species-specific stimulation of growth by NIR.

Growth of *R. mucilaginosa* strains F-J1 (A), DSM 13621 (B) and DSM 18184 (C), and *S. cerevisiae* W303 (D) was performed under 880 nm NIR irradiation at 20 °C. (E) Comparison of cell counts in *S. cerevisiae* expressing MRPS18b after 20 hours of cultivation in induction medium under 940 nm irradiation or 880 nm irradiation at 25 °C.

Table S1.

Pairwise comparisons (Tukey HSD) of growth rates at significant temperatures.

| **Temperature** | **Comparison** | **Mean Diff** | **p-value** | **Sig.** |
| --- | --- | --- | --- | --- |
| 15 °C | 880nm vs 940nm | 0.01173 | 0.2879 | ns |
|  | 880nm vs Dark | 0.05014 | < 0.001 | **** |
|  | 940nm vs Dark | 0.03841 | 0.0037 | ** |
| 20 °C | 880nm vs 940nm | -0.01712 | 0.4653 | ns |
|  | 880nm vs Dark | 0.06699 | 0.0063 | ** |
|  | 940nm vs Dark | 0.08411 | 0.0020 | ** |
| 25 °C | 880nm vs 940nm | -0.05267 | 0.0042 | ** |
|  | 880nm vs Dark | 0.10321 | < 0.001 | **** |
|  | 940nm vs Dark | 0.15588 | < 0.001 | **** |

Table S2.

Significantly different proteins between IR 880nm and dark conditions in quantitative proteomic analysis. Significance criteria: log_2_(fold change) > 1 (IR 880 nm ‘upregulated’ or dark ‘downregulated’) or log_2_(fold change) < -1 (IR 880 nm ‘downregulated’, dark ‘upregulated’), and p-value < 0.1. Fold change = IR 880 nm / dark. n = 5 biological replicates per condition.

| **Protein ID** | **log_2_(fold change)** | **Fold Change** | **p-value** | **Primary Function** | **Functional Category** |
| --- | --- | --- | --- | --- | --- |
| FJ10461 | +2.75 | 6.73 | 0.001349 | Mitochondrial ribosomal protein S18 | Protein synthesis |
| FJ12642 | +2.24 | 4.72 | 0.083176 | Unknown function (DUF202 domain) | Unknown function |
| FJ15279 | +2.03 | 4.08 | 0.008128 | Ubiquitin carboxyl-terminal hydrolase | Protein degradation |
| FJ12989 | +1.50 | 2.83 | 0.054954 | BAG domain protein | Molecular chaperone |
| FJ14950 | +1.11 | 2.16 | 0.056234 | Regulatory subunit of dimeric UBA3-ULA1 E1 enzyme | Ubiquitination |
| FJ16920 | -3.60 | 12.12 | 0.005495 | Unknown function | Unknown function |
| FJ11308 | -2.10 | 4.29 | 0.013183 | Heme-degrading enzyme | Heme metabolism |
| FJ14978 | -1.94 | 3.84 | 0.041687 | Lysine methyltransferase | Epigenetic regulation |
| FJ12392 | -1.80 | 3.48 | 0.072444 | Mitochondrial import protein PAM17 | Protein transport |
| FJ11207 | -1.71 | 3.27 | 0.027542 | Alcohol dehydrogenase GroES-like domain | Alcohol metabolism |
| FJ11323 | -1.68 | 3.20 | 1.07E-05 | SUZ domain protein | Transcriptional regulation |
| FJ13352 | -1.43 | 2.69 | 0.00537 | RNA recognition motif | RNA processing |
| FJ12089 | -1.41 | 2.65 | 0.01122 | Set domain protein | Epigenetic regulation |
| FJ12356 | -1.37 | 2.58 | 0.075858 | Aldolase/RraA | Sugar metabolism |
| FJ16747 | -1.30 | 2.46 | 0.005248 | Rab subfamily small GTPase | Membrane transport |
| FJ12188 | -1.29 | 2.44 | 0.001 | Mannitol-1-phosphate dehydrogenase | Alcohol metabolism |
| FJ15111 | -1.21 | 2.31 | 0.079433 | GLE1-like protein | Nucleocytoplasmic transport |
| FJ14327 | -1.20 | 2.30 | 0.010715 | Methylenetetrahydrofolate reductase | Methionine metabolism |
| FJ16824 | -1.14 | 2.20 | 0.000513 | Unknown function (DUF952 domain) | Unknown function |
| FJ14525 | -1.11 | 2.16 | 0.039811 | ChAPs protein (Chs5p-Arf1p-binding protein) | Cell cycle |
| FJ10607 | -1.11 | 2.16 | 0.007244 | Glutathione S-transferase N-terminal domain | Detoxification |
| FJ15104 | -1.07 | 2.10 | 0.064565 | Carboxyltransferase domain | Metabolic enzyme |
| FJ15262 | -1.03 | 2.04 | 0.019953 | Acyl-CoA dehydrogenase N-terminal domain | Fatty acid metabolism |

Table S3.

Ribosomal proteins S18 used in phylogenetic study.

| **Abbreviation** | **Species** | **Strain** | **Protein** | **Accession** |
| --- | --- | --- | --- | --- |
| FJ1_MRPS18a | *R. mucilaginosa* | F-J1 | MRPS18a | NODE_3_length_1398165_cov_30.075783.g1446 |
| FJ1_MRPS18b | *R. mucilaginosa* | F-J1 | MRPS18b | NODE_1_length_1989854_cov_32.229966.g375 |
| FJ1_RPS18a/b | *R. mucilaginosa* | F-J1 | RPS18a | NODE_1_length_1989854_cov_32.229966.g303 |
| FJ1_RPS18a/b | *R. mucilaginosa* | F-J1 | RPS18b | NODE_32_length_74737_cov_35.713113.g6622 |
| JCM8115_MRPS18a | *R. mucilaginosa* | JCM8115 | MRPS18a | GCA_049500115.1 |
| JCM8115_MRPS18b | *R. mucilaginosa* | JCM8115 | MRPS18b | GCA_049500115.1 |
| JCM8115_RPS18a/b | *R. mucilaginosa* | JCM8115 | RPS18a/b | GCA_049500115.1 |
| CYJ03_MRPS18a | *R. mucilaginosa* | CYJ03 | MRPS18a | GCA_004143675.1 |
| CYJ03_MRPS18b | *R. mucilaginosa* | CYJ03 | MRPS18b | GCA_004143675.1 |
| CYJ03_RPS18a/b | *R. mucilaginosa* | CYJ03 | RPS18a/b | GCA_004143675.1 |
| GDMCC_MRPS18a | *R. mucilaginosa* | GDMCC2.30 | MRPS18a | GCA_034406715.1 |
| GDMCC_MRPS18b | *R. mucilaginosa* | GDMCC2.30 | MRPS18b | GCA_034406715.1 |
| GDMCC_RPS18a/b | *R. mucilaginosa* | GDMCC2.30 | RPS18a/b | GCA_034406715.1 |
| JGTA-S1_MRPS18a | *R. mucilaginosa* | JGTA-S1 | MRPS18a | GCA_003055205.1 |
| JGTA-S1_MRPS18b | *R. mucilaginosa* | JGTA-S1 | MRPS18b | GCA_003055205.1 |
| JGTA-S1_RPS18a/b | *R. mucilaginosa* | JGTA-S1 | RPS18a/b | GCA_003055205.1 |
| JY1105_MRPS18a | *R. mucilaginosa* | JY1105 | MRPS18a | GCA_024748845.1 |
| JY1105_MRPS18b | *R. mucilaginosa* | JY1105 | MRPS18b | GCA_024748845.1 |
| JY1105_RPS18a/b | *R. mucilaginosa* | JY1105 | RPS18a/b | GCA_024748845.1 |
| LBMH_MRPS18a | *R. mucilaginosa* | LBMH1012 | MRPS18a | GCA_024500305.1 |
| LBMH_MRPS18b | *R. mucilaginosa* | LBMH1012 | MRPS18b | GCA_024500305.1 |
| LBMH_RPS18a/b | *R. mucilaginosa* | LBMH1012 | RPS18a/b | GCA_024500305.1 |
| Sacc_MRPS18a | *S. cerevisiae* | S288C | MRPS18a | NP_014093.1 |
| Sacc_RPS18a | *S. cerevisiae* | S288C | RPS18a | NP_010738.1 |
| Sacc_RPS18b | *S. cerevisiae* | S288C | RPS18b | NP_013686.1 |
| Homs_MRPS18c | *Homo sapiens* | / | MRPS18b1 | 6NU2_AP |
| Homs_MRPS18a | *Homo sapiens* | / | MRPS18b2 | NP_057151.1 |
| Homs_MRPS18b | *Homo sapiens* | / | MRPS18b3 | NP_001284696.1 |
| Homs_RPS18a | *Homo sapiens* | / | RPS18a | NP_072045.1 |
| Homs_RPS18b | *Homo sapiens* | / | RPS18b | 8OZ0_n |
| Homs_RPS18c | *Homo sapiens* | / | RPS18c | 8G5Z_SS |
| Homs_RPS18d | *Homo sapiens* | / | RPS18d | 6OLE_SS |

SI References

1.	Qian XX, Santini CL, Kosta A, Menguy N, Le Guenno H, Zhang W *et al.*, Juxtaposed membranes underpin cellular adhesion and display unilateral cell division of multicellular magnetotactic prokaryotes. *Environ Microbiol* 2020;22**(4)**:1481-94.

2.	Dai J, Li XG, Zhang TY, Chen H, Zhang WJ, Li DH *et al.*, Illuminating a bacterial adaptation mechanism: Infrared-driven cell division in deep-sea hydrothermal vent environments. *The Innovation Geoscience* 2024;2**(1)**:100050.

3.	Wright R. Transmission electron microscopy of yeast. *Microsc. Res. Tech* 2000;51**(6)**:496-510.

4.	Reynolds ES. The use of lead citrate at high pH as an electron-opaque stain in electron microscopy. *J Cell Biol* 1963;17**(1)**:208-12.

5.	Conrad R, Narayan K. Instance segmentation of mitochondria in electron microscopy images with a generalist deep learning model trained on a diverse dataset. *Cell Syst* 2023;14**(1)**:58-71.e5*.*

6.	Chen S, Zhou Y, Chen Y, Gu J. fastp: an ultra-fast all-in-one FASTQ preprocessor. *Bioinformatics* 2018;34**(17)**:i884-i890.

7.	Bankevich A, Nurk S, Antipov D, Gurevich AA, Dvorkin M, Kulikov AS, et al. SPAdes: A new genome assembly algorithm and its applications to single-cell sequencing. *J Comput Biol* 2012;19**(5)**:455-77.

8.	Alonge M, Soyk S, Ramakrishnan S, Wang X, Goodwin S, Sedlazeck FJ, et al. RaGOO: Fast and accurate reference-guided scaffolding of draft genomes. *Genome Biol* 2019;20**(1)**:224.

9.	Brůna T, Lomsadze A, Borodovsky M. GeneMark-EP+: Eukaryotic gene prediction with self-training in the space of genes and proteins. *NAR Genom Bioinform* 2020;2**(2)**:lqaa026.

10.	Cantalapiedra CP, Hernández-Plaza A, Letunic I, Bork P, Huerta-Cepas J. eggNOG-mapper v2: Functional annotation, orthology assignments, and domain prediction at the metagenomic scale. *Mol Biol Evol* 2021;38**(12)**:5825-9.

11.	Tillich M, Lehwark P, Pellizzer T, Ulbricht-Jones ES, Fischer A, Bock R, et al. GeSeq – versatile and accurate annotation of organelle genomes. *Nucleic Acids Res* 2017;45**(W1)**:W6-W11.

12.	Tyanova S, Cox J. Perseus: A bioinformatics platform for integrative analysis of proteomics data in cancer research. In: von Stechow L, ed. Cancer Systems Biology: Methods and Protocols. Methods in Molecular Biology, vol 1711. New York: Humana Press; 2018. pp. 133-148.

13.	Lin J, Zhou D, Steitz TA, Polikanov YS, Gagnon MG. Ribosome-targeting antibiotics: Modes of action, mechanisms of resistance, and implications for drug design. *Annu Rev Biochem* 2018;87:451-78.
